# Influence of the Framing Effect, Anchoring Effect, and Knowledge on Consumers’ Attitude and Purchase Intention of Organic Food

**DOI:** 10.3389/fpsyg.2020.02022

**Published:** 2020-08-12

**Authors:** Lijie Shan, Haimeng Diao, Linhai Wu

**Affiliations:** ^1^Institute for Food Safety Risk Management, School of Business, Jiangnan University, Wuxi, China; ^2^School of Business, Jiangnan University, Wuxi, China

**Keywords:** organic food, framing effect, anchoring effect, consumers’ attitude, purchase intention, product knowledge

## Abstract

This article explores consumers’ attitude toward and purchase intention of organic food regarding the influence of the framing effect and anchoring effect and the role of knowledge. Our findings suggest that whether message framing describes the benefits of buying organic food or the loss resulting from a failure to buy organic food, significantly influences consumers’ attitude and purchase intention. In addition, presenting an anchor price in advertisements also significantly influences consumers’ judgment. These results indicate that a negatively framed message induces a more favorable attitude and purchase intention than a positively framed message, a low anchor price is more favorable than a high one, and the interaction effect of framing and anchoring is not significant at the 1% level. Finally, consumers with less organic food knowledge are more susceptible to framing and anchoring effects. These results provide suggestions for appropriate message framing and price anchoring to enhance consumption within the organic industry.

## Introduction

Rapid growth in industrial development and improved living standards are increasing consumers’ awareness of food safety and their desire to consume healthy and environmentally sustainable foods. Organic foods undergo a stringent certification process and are produced without the application of synthetic chemicals, such as fertilizers and pesticides (Basha et al., 2015). To this end, the attention paid to organic foods is increasing.

According to the International Federation of Organic Agriculture Movements (IFOAM), globally, organic agricultural land is growing at a rate of 20% per year. However, despite the global growth in production, the market for organic goods is still relatively small. Only 1.4% of agricultural land in the world is farmed organically and, for 56% of countries where data is available, less than 1% of their total farmland is organic farmland (FiBL and IFOAM-Organics International, 2019). In China, organic food only accounts for 0.6% of the domestic food market, and annual per capita consumption is less than $6. This is lower than the world average and far from the amount spent per capita in developed countries.

There are several reasons for the low consumption of organic food, including consumers’ attitudes and purchase intentions. Most of the existing literature focus on the factors that influence these aspects, including consumers’ product knowledge (Wu et al., 2019), trust (Yue et al., 2017), health awareness, and individual characteristics (Asioli et al., 2017) on the basis of the Theory of Reasoned Action (TRA) and the Theory of Planned Behavior (TPB) (Zagata, 2012). However, to the best of our knowledge, no extant study has used the framing and anchoring perspectives for exploring ways to encourage consumers’ positive responses.

Consumers today come across a variety of information when they browse available products. The information delivered through labels mainly includes product advertising messages and price information cues (Wu and Cheng, 2011). For example, Levin and Gaeth (1988) presented an advertisement for ground beef to two groups: one was framed as “75% lean” and the other as “25% fat.” Participants responded more favorably toward the beef when it was described as 75% lean. Other studies have examined the effect of including the price of alternative products, label prices, and other information as “anchor value” (Chandrashekaran and Grewal, 2006; Rödiger and Hamm, 2015). For products of the same quality, consumers prefer products that are advertised as having a lower price compared to an internal reference price. The anchor price changes consumers’ attitudes and purchase intentions (Chang and Wu, 2015; Paswan et al., 2016). Consumers tend to find “satisfactory solutions” using heuristic strategies and process information by identifying, editing, and evaluating based on their own product knowledge rather than relying on “optimal solutions” (Dale, 2015). Thus, individual decision-making can be influenced by the way information is presented and consumers with more product knowledge can process information more effectively and choose more suitable products (Wang et al., 2019). Therefore, consumers are often influenced by the message framing and anchor pricing in advertisements. Studies have examined framing and anchoring effects in investing, charitable donations, and consumption decisions (Levin et al., 2002; Sinha and Adhikari, 2017). However, consumers’ attitude and purchase intention toward organic food based on the framing and anchoring effects has not been studied.

Therefore, this article applies framing and anchoring effects to study organic food consumers’ attitude and purchase intention and investigats the moderation role of consumer’s product knowledge on these two categories. Based on the findings in this study, it provides suggestions for adjusting consumers’ attitudes and purchase intentions, increasing organic food consumption, developing potential markets for organic food, and developing the organic industry.

## Literature Review and Hypotheses

According to the theory of bounded rationality (Simon, 1955), consumers do not analytically edit external information on products and cannot perform subtle estimations due to their limited knowledge and uncertainty. Instead, consumers use heuristic systems to identify, edit, and make intuitive judgments based on their knowledge of a given product (Li and Ding, 2010; Shan et al., 2019). Consumers’ attitudes and purchase intentions, therefore, are influenced by their product knowledge, advertising messages, prices, and the limitations of their information processing (Hoque et al., 2018). Specifically, more knowledgeable consumers tend to develop a better cognitive structure to process information effectively, while consumers with less product knowledge usually make biased judgments because of their limited experience (Bettman and Park, 1980). It is likely that consumers who are more knowledgeable about organic food are less likely to be influenced by framing and anchoring effects than those who with less knowledge.

### Framing Effect

“Message framing” is a communication strategy used to influence judgment, attitude, and behavior through equivalent appeals, framed as the benefits gained or consequences incurred from buying a product (Levin et al., 1998). Negatively framed messages emphasize the undesirable consequences of refusing to buy a product or service, whereas positively framed messages emphasize the desirable profit or benefits of buying a product or service. Previous studies on message framing have shown mixed results: some indicate that positively framed messages are more persuasive (Van de Velde et al., 2010), while others find that negatively framed messages have greater power to enhance information processing and promote consumers’ attitude and purchase intention. For example, Moon et al. (2016) examined bio-fuels, finding that highlighting the negative impact of gasoline use is most effective in increasing consumers’ biofuel purchase intention. Likewise, Chen (2016) found that emphasizing the benefits of purchasing health care products is less convincing than emphasizing the loss of not purchasing the products. Therefore, loss aversion makes the negative frame more persuasive.

Message framing has a large impact on consumers’ attitudes and purchase intentions; thus, the advertising message framing is crucial (Block and Keller, 1995; Zhu, 2014). This study used positively framed messages that suggest the environmental and personally benefit gained from purchasing organic food. The negatively framed message, meanwhile, emphasizes that consumers may cause environmental damage and incur personal losses if they do not purchase organic food. Although both convey information to induce favorable attitudes and purchase intentions (Martins et al., 2019), the extent of their impact may differ. The following hypotheses seek to explore this difference:

H1a: Respondents facing a negatively framed message will form a more positive attitude toward organic food than those facing a positively framed message.
H1b: Respondents facing a negatively framed message will be more likely to purchase organic food than those facing a positively framed message.

Knowledge of organic food reflects consumers’ understanding of organic food concepts and corresponding attributes, while subjective knowledge reflects self-evaluation and consumers’ ability to process information, and can effectively predict consumer behavior (Teng and Wang, 2015). High knowledge levels improve the effectiveness and accuracy of consumers’ information processing and help form stable consumer preferences and purchase intentions (Cai et al., 2016). Nelson et al. (1997) argue that participants with higher levels of professional knowledge actively compare different message frames and weigh the reliability of information, thereby strengthening the framing effect. However, other studies found that consumers’ existing knowledge promotes information processing and weakens the framing effect and that consumers with less knowledge have less credible opinions and are more likely to make judgments based on incomplete experience and insufficient information processing, meaning they are more susceptible to the influence of the framing effect (Kinder and Sanders, 1990). Increased consumer product knowledge, therefore, should weaken the framing effect and decrease bias in consumer attitudes and purchase intentions (Haider-Markel and Joslyn, 2001; Jin and Han, 2014). This leads to another hypothesis:

H1c: Respondents with less knowledge about organic food are more susceptible to the framing effect.

### Anchoring Effect

Tversky and Kahneman (1974) were the first to propose the anchoring effect. They suggest that consumers are not always rational when making decisions, often adjusting their estimates based on prior knowledge and reference information by the anchoring and adjustment heuristic. As a result, anchor value is an important factor. The anchoring effect is a robust idea that has been verified in different domains, including economic decision-making (Oechssler et al., 2009), value evaluation (Chang et al., 2016), and bank lending (Dougal et al., 2015). Here, the subjects are accustomed to an adjustment process to make their estimates, but if they face a low anchor, the final estimates will be lower than those of someone who began with a high anchor (Tversky and Kahneman, 1974; Northcraft and Neale, 1987).

In general, when there is uncertainty about a product, consumers are prone to form their attitudes and purchase intentions according to accessible information, such as advertising prices. For example, Santosh and Mrinalini (2015) found that the last number of the label price plays an important role in consumers’ behavior. Moreover, Shen et al. (2016) suggest that higher external price information for other goods presented in the decision-making environment increases consumers’ acceptance of actual prices, so consumer attitudes and purchase intentions will be more favorable. However, organic food in domestic China is still in the primary period of development, and is, therefore, barely known to general consumers. Also, the price of organic food is 3–5 times—and sometimes even 8–10 times greater than the price of non-organic food in China (Certification, and Accreditation Administration of the People’s Republic of China, 2014). As a result of unfamiliarity with and limited knowledge about organic food, consumers use other prices as their internal reference point, such as the price of conventional, non-organic food (Lin and Chen, 2017). Such consumers find conventional food for a lower price than the organic label price, leading to a feeling of deception and unfairness toward the external anchor price (Weisstein et al., 2016). This affects consumers’ perceived benefits and results in a different anchoring effect (Niedrich et al., 2001; Rödiger and Hamm, 2015). Thus, it is reasonable to argue that general consumers will form more negative attitudes and lower purchase intentions toward organic food when they are presented with the high anchor prices. This leads to the following hypotheses:

H2a: Respondents facing a low anchor price for organic food will have a more positive attitude than those given a high anchor price.
H2b: Respondents facing a low anchor price for organic food will be more likely to purchase organic food than those given a high anchor price.

The role of knowledge in anchoring effect investigations has different results depending on the domains (Englich, 2008). For example, Northcraft and Neale (1987) demonstrate that the anchoring effect is moderated by participates’ knowledge. Although both participants with and without related knowledge are influenced by anchoring effects, the anchoring effect’s influence is less on respondents who are more well-informed compared to those who are less. Zhang and Zhao (2016), meanwhile, report that respondents’ familiarity with risk also affects the anchoring effect; the less familiar someone is with a product, the more prone they are to judgment biases based on different anchor values. Consumers’ behavior is no exception. Consumers with a high degree of product knowledge are more accurate and confident in their estimation, consequently influencing their attitudes and purchase intentions (Biswas and Sherrell, 1993). Therefore, it is reasonable to hypothesize:

H2c: Respondents with less knowledge about organic food are more susceptible to the anchoring effect.

## Materials and Methods

### Message Framing and Anchor Price

Based on the research of Grewal et al. (1994) and Chang (2007), this study adopted positively and negatively framed messages for organic food advertisements. It positively framed organic lettuce by saying:

Organic food uses natural and ecological production methods, not only providing you with safe food but also fostering sustainable environmental development, thereby benefiting everyone. When you decide to purchase organic lettuce, you are making a healthy decision that also protects the environment. There is no doubt that there are many benefits to purchasing and eating organic lettuce. By choosing organic food, you are consuming lettuce that is free of harmful content such as chemicals, antibiotics, and pesticides. Choosing Organic lettuce is not only an advantage for your health but also reduces your impact on the environment. It is good for everyone.

For negative framing, it described the same product using the following phrasing:

Organic food uses natural and ecological production methods, not only providing you with safe food but also fostering sustainable environmental development. When you decide against purchasing organic lettuce, you are making an unhealthy decision and harm the environment. Obviously, there are many disadvantages to purchasing and eating non-organic lettuce. By choosing non-organic lettuce, you are consuming lettuce that contains high levels of harmful content, such as chemicals, antibiotics, and pesticides. Choosing non-organic not only harms your health but also increases your negative impact on the environment. It is nothing but harmful.

To determine the anchor price, this study relied on the work of Chapman et al. (2002). Because there is a significant anchoring effect when a respondent pays more attention to the “anchor value,” it used the label price of the organic lettuce as the anchor value. Using Jacowitz and Kahneman’s (1995) concept of the external anchoring index (AI), the quintiles of 85 and 15% of the estimation in the control group acted as high and low anchor values in the test groups, respectively. Responses to a pre-survey administered to the control group where participants were asked to estimate the price of the organic lettuce, determined the 85% (15 RMB) and 15% quantiles (3 RMB).

### Experimental Design

There are two questionnaires for the control group and four for the test groups. In the control group, surveyed consumers were asked to estimate the price of organic lettuce with different message frames and without related price information. In contrast, respondents in the test groups were given 15 (high) or 3 (low) RMB as the price anchor and then instructed to make respective judgments using a positively or negatively framed message.

After conducting a pre-survey in Wuxi, Jiangsu Province, China, the questionnaires underwent revision to ensure validity. The pilot study was conducted in January 2019 with a random sample of 60 respondents (24 males and 36 females). Of these, three had difficulty judging the price of organic lettuce giving no suggestions and did not answer the question regarding price. Excluding these, the average estimated price was 7.4 RMB, which is less than the market price. The pre-survey suggested that respondents could not seriously form attitude and purchase intention without price information. As a result, the final survey used high and low anchor prices alongside positively and negatively framed messages to investigate the anchoring and framing effects.

There were three parts to each questionnaire. The first part addressed the respondents’ knowledge of organic food. The second provided respondents with information about organic lettuce, including price and advertising message, and the third measured the respondents’ attitudes toward organic lettuce and their purchase intention.

### Experimental Organization

All interviewers were from the Institute for Food Safety Risk Management at Jiangnan University. We recruited 400 respondents by selecting every third consumer (Wu et al., 2012) from five administrative districts of Wuxi, who were randomly assigned to one of the four groups to ensure the representativeness of the sample. This also ensured that each consumer had an equal chance to be chosen and improved the fit of the sample to the whole group.

The formal survey was carried out between June 5–20, 2019. The interviewers collected a total of 368 valid questionnaires, including 92 from Group 1 (low anchor × positive framing), 92 from Group 2 (low anchor × negative framing), 93 from Group 3 (high anchor × positive framing), and 91 from Group 4 (high anchor × negative framing). Each respondent who completed the survey received RMB 5 in compensation.

Of the total respondents, 37.2% of them were male, 74.5% had a college-or university-level education, and 57.6% were aged 26–55 years old. Thirty-four percent were low-income individuals (annual income of 36,000 RMB or less). In addition, most respondents agreed that they were in good health. Differences in demographics between groups were examined using χ^2^ tests. The results indicate no significant differences among any demographic variables (Table 1).

**TABLE 1:** Demographic characteristic of participants.

**Demographics**	**Group 1**	**Group 2**	**Group 3**	**Group 4**	**Total (%)**	***P-*value**
**Gender**						
Male	35	39	28	35	37.2	0.371
Female	57	53	65	56	62.8	
**Age**						
18–25	33	41	33	36	38.9	0.271
26–35	33	34	33	28	34.8	
36–45	19	9	13	21	16.8	
46–55	5	4	8	5	6.0	
56–65	2	4	6	1	2.2	
**Marital status**						
Married	44	49	41	50	50.0	0.433
Unmarried	48	43	52	41	50.0	
**Education**						
Less than junior college	25	25	30	17	25.6	0.858
Junior college	26	21	18	30	25.8	
Higher than junior college	41	47	47	44	48.7	
**Annual income**						
36,000 RMB and less	33	30	37	25	34.0	0.567
36,000–50,000 RMB	9	16	14	11	13.6	
50,000–80,000 RMB	16	12	12	23	17.1	
80,000–10,000 RMB	17	18	13	14	16.8	
More than 100,000	17	16	17	18	18.5	
**Health status**						
Healthy	80	84	73	76	85.1	0.475
Moderately healthy	12	7	18	15	14.1	
Unhealthy	0	1	2	0	0.8	

### Validity and Reliability

This study used SPSS 20.0 and AMOS 7.0 to test the reliability and validity of the questionnaire based on scales in prior studies (Kim et al., 2008; Cucchiara et al., 2015; Konuk, 2018). It measured consumers’ attitude and purchase intention toward organic food using a 7-point Likert scale. Higher scores revealed a stronger purchase intention and a more positive attitude. Similarly, consumers’ knowledge about organic food was scored on a 5-point Likert scale (1 = good understanding, 5 = ignorant), with a low score displaying higher knowledge.

Table 2 shows the reliability of each item using Cronbach’s alpha. The values were 0.73 (knowledge), 0.86 (attitude), and 0.94 (intention). These reliability coefficients are all higher than the critical value of 0.70, suggesting high internal reliability (Fornell and Larcker, 1981).

**TABLE 2:** Validity and reliability of study variables.

**Variables**	**Latent variables**	**Factor loadings**
Product Knowledge Cronbach’s alpha = 0.73	PK1: Compared to others, how knowledgeable do you think you are with organic food?	0.709
	PK2: Do you know how to distinguish organic food?	0.738
	PK3: Do you think you can purchase organic food satisfactorily based on only your own knowledge?	0.628
Attitude Cronbach’s alpha = 0.86	A1: Organic food is extremely bad-extremely good.	0.778
	A2: Organic food is extremely unhealthy-extremely healthy.	0.845
	A3: Organic food is extremely unattractive-extremely attractive.	0.823
Purchase Intention Cronbach’s alpha = 0.94	PI1: I will purchase this organic lettuce even if I have already purchase one.	0.909
	PI2: I tend to purchase this organic lettuce.	0.930
	PI3: I will suggest my friends to purchase this organic lettuce.	0.913

Discriminant validity, showing the degree of constructs measured in different methods, is distinguishable (Fornell and Larcker, 1981). One principle for discriminant validity is that the correlation coefficient between one construct and the others should be less than the square root of the average variance extracted (AVE) for each variable. The diagonal of Table 3 shows the AVE square roots, all of which are greater than the correlation coefficient, indicating a favorable discriminant validity.

**TABLE 3:** Average variance extracted and correlation of constructs.

**Variables**	**AVE**	**Product knowledge**	**Attitude**	**Purchase intention**
Product knowledge	0.48	0.693*		
Attitude	0.67	0.067	0.816*	
Purchase intention	0.84	0.048	0.735	0.917*

* The diagonal row numbers are square roots of the AVE. Off–diagonal numbers are the correlations among variables.

Averagevarianceextracted(AVE)=(∑standardized

(1) $${\text{loading}}^{2}/\left[\left(\mathrm{standardized}{\mathrm{loading}}^{2}\right)+\sum \varepsilon_{\text{j}}\right])$$

## Results and Discussion

We used analysis of variance (ANOVA) to test the framing and anchoring effect on consumers’ attitude and purchase intention toward organic food. Further, we examined the role of consumers’ knowledge using hierarchical multiple regression (HMR).

### The Main Effect of Framing and Anchoring

The ANOVA results reveal that framing and anchoring had no significant interaction effect on consumer’s attitude and intention (*F*_attitude_ = 0.752, *P* > 0.01, *F*_intention_ = 4.582, *P* > 0.01). The *R*^2^ of attitude and intention are 0.315 and 0.325, indicating a main effect construct of 31.5 and 32.5%, respectively (Table 4).

**TABLE 4:** Results of ANOVA test.

**Manipulation**	***N***	**Attitude**		**Purchase intention**	
		**Mean**	***F***	**Mean**	***F***
Negatively framed message	183	4.461	61.430***	4.031	56.728***
Positively framed message	185	3.580		3.096	
Low anchor price	184	4.591	104.516***	4.219	113.629***
High anchor price	184	3.446		2.902	
Framing × anchoring	368	4.018	0.752	3.561	4.582**

$R_{a\,t\,t\,i\,t\,u\,d\,e}^{2}=0.315;R_{i\,n\,t\,e\,n\,t\,i\,o\,n}^{2}=0.325$. *** *p* < 0.001, ** *p* < 0.05.

Moreover, for the different message frames and anchor prices, the significance of the *F* statistics of consumer attitudes and purchase intentions are less than 0.01. This indicates that the main framing and anchoring effect on consumers’ attitude and purchase intention of organic food was significant. Specifically, the consumers exposed to the negatively framed message rated the organic lettuce more positively (*M*_attitude_ = 4.461) and had a higher intention to purchase (*M*_intention_ = 4.031) than those exposed to the positively framed message (*M*_attitude_ = 3.580, *M*_intention_ = 3.096). These results are consistent with those of previous studies (Maheswaran and Meyers-Levy, 1990). Negative framing promotes deep processing of information and improves persuasiveness, so it is possible that the consumers’ tendency toward loss aversion makes the potential loss of not buying organic lettuce more unacceptable.

In addition, the consumers exposed to a low anchor price rated the organic lettuce more positively (*M*_attitude_ = 4.591) and revealed a higher purchase intention (*M*_intention_ = 4.219) than those exposed to a high anchor price (*M*_attitude_ = 3.446, *M*_intention_ = 2.902). This finding is inconsistent with those of previous studies, such as those by Shen et al. (2016). This may be because the anchor price for their study was based on the reference price of a counterpart food, in this case, a high anchor value is conducive to increasing the consumer’s recognition of the target product. In the present study, however, the high anchor price highlights the gap between organic and conventionally produced lettuce, resulting in a strong contrast effect and reducing consumer acceptance and purchase intention toward organic food. This is consistent with the findings of Wilson et al. (1996) in that there is a significant anchoring effect on consumers’ attitude and purchase intention and that low anchor price information can improve consumers’ attitude and increase their purchase intention. Thus, Hypotheses H1a and H1b and Hypotheses H2a and H2b are confirmed.

### The Role of Product Knowledge in Framing Effect and Anchoring Effect

The study went on to examine the role of product knowledge on framing and anchoring effects by conducting HMR models.

**FIGURE 1:**
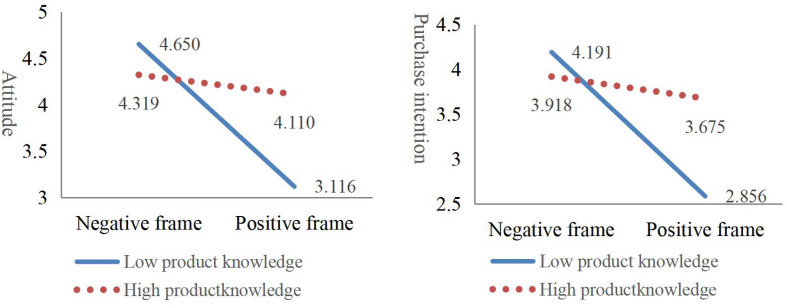
Respondents’ responses in different message framing.

The results shown in Table 5 and Figure 1 are clear; the interaction coefficients of the message frame and product knowledge (β_attitude_ = 0.332, *P* < 0.01 and β_purchase intention_ = 0.341, *P* < 0.01) indicate that they have a positive influence on attitude and purchase intention. When categorized based on their knowledge level according to their survey scores, more knowledgeable consumers were less likely to change their attitudes or purchase intentions based on the message frame (Figure 1).

**TABLE 5:** Hierarchical multiple regression results for framing effect.

	**β**					
**Models**	**Attitude**			**Purchase intention**		
Gender	–0.064	0.006	–0.018	–0.166	–0.088	–0.113
Age	–0.105	–0.072	–0.018	–0.145	–0.110	–0.055
Marital status	0.224	0.291*	0.229	0.309	0.381**	0.317*
Education	−0.167**	−0.159**	−0.141**	−0.210*	−0.203***	−0.185***
Health	−0.187*	−0.179**	−0.145*	−0.192*	−0.182*	–0.147
Income	0.026	–0.001	–0.017	–0.023	–0.052	–0.069
*X*_1_		−0.435***	−0.436***		−0.461***	−0.463***
*M*		0.150**	0.168***		0.188***	0.206***
*X*_1_ × *M*			0.332***			0.341***
△*R*^2^	0.035	0.131	0.063	0.047	0.125	0.054
△*F*	2.205**	28.242***	29.440***	2.934**	27.082***	24.972***

*X*_1_ = message frame, *M* = product knowledge. *** *p* < 0.001, ** *p* < 0.05, * *p* < 0.1.

These results indicate that those with more knowledge were less influenced by the framing effect. This finding is inconsistent with those of Bullock and Vedlitz (2017). One reason for this is the difference in subject area; Bullock and Vedlitz (2017) examined controversial public policies. People who know little about public policies are indifferent and thus do not respond strongly to the framing effect. However, food safety is closely related to consumers’ daily lives. Concerns about food safety prompt many to pay attention to product information, so consumers who have less knowledge of organic food will rely on external message frames, thereby creating a stronger framing effect. This finding confirms Hypothesis H1c.

**FIGURE 2:**
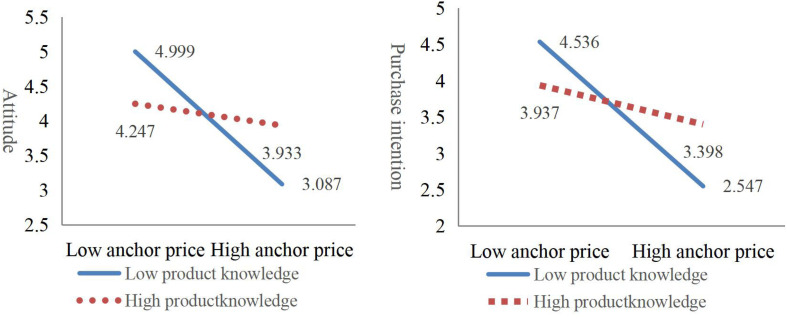
Respondents’ responses in different anchor price.

To analyze the role product knowledge plays in the anchoring effect, this study also involved developing HMR models (Table 6). The interaction coefficient between anchor price and knowledge—β_attitude_ = 0.400 (*P* < 0.01) and β_purchase intention_ = 0.363 (*P* < 0.01)—indicates the interaction between attitude and purchase intention. Consumers with a high knowledge level are less likely to change their attitudes or purchase intentions at different anchor prices, indicating that those with more product knowledge are less affected by the anchoring effect (Figure 2). This aligns with Englich et al. (2016) who found that consumers with a deeper understanding of organic food can edit price cues based on their own product knowledge, generate spontaneous anchors, reduce the impact of external anchors, and reduce the anchoring effect. This confirms Hypothesis H2c.

**TABLE 6:** Hierarchical multiple regression results for anchoring effect.

	**β**					
**Models**	**Attitude**			**Purchase intention**		
Gender	–0.064	0.032	0.022	–0.166	–0.055	–0.064
Age	–0.105	–0.055	–0.055	–0.145	–0.088	–0.088
Marital status	0.224	0.198*	0.169	0.309	0.28**	0.254*
Education	−0.167**	−0.136**	−0.132**	−0.210*	−0.177***	−0.172***
Health	−0.187*	−0.148**	−0.131*	−0.192*	−0.147*	–0.132
Income	0.026	0.017	–0.040	–0.023	–0.034	–0.046
*X*_2_		−0.545***	−0.557***		−0.622***	−0.633***
*M*		0.120**	0.024***		0.151***	0.063***
*X*_2_ × *M*			0.400***			0.363***
△*R*^2^	0.035	0.194	0.089	0.047	0.207	0.059
△*F*	2.205**	45.190***	46.700***	2.934**	46.690***	30.783***

*X*_2_ = anchor price. *** *p* < 0.001, ** *p* < 0.05, * *p* < 0.1.

## Conclusion

This study shows significant framing and anchoring effects in consumers’ attitude and purchase intention toward organic food. With a non-significant, 1% level interaction effect between them, the framing and anchoring effects can be replicated in the consumption of organic food, an area neglected in prior research. The results of this work suggest that a negatively framed message and a low anchor price enhances the persuasion of advertisements in relation to consumer responses regarding attitudes and purchase intentions. Further, this paper confirms the moderating role of product knowledge on framing and anchoring effects, demonstrating that less knowledgeable consumers are more susceptible to both effects.

Improving consumers’ purchase intention and attitude toward organic food is critical to long-term consumption. Our results suggest that the government should take advantage of the internet, television advertisements, and other media to educate the public on health problems caused by pesticide residues and antibiotics in much of the food supply, emphasizing through a negatively framed message, that these chemicals may cause health problems. They should explain to supermarkets and organic farms how organic certification may improve consumers’ knowledge to enabling them to identify, purchase, and consume organic food, thus making healthy consumption choices. In addition, our results indicate that consumers’ attitudes and purchasing intentions are significantly lower when organic food has a high anchor price. Therefore, the government should increase support for the organic industry, provide appropriate organic facilities, organic conversion subsidies, organic certification subsidies and input subsidies for organic production enterprises to lower the production cost of organic food gradually. Companies should lower the circulation costs of organic food and decrease the price gap between organic and other foods, thereby improving consumers’ attitude and purchase intention.

There are a few limitations to this study. First, past studies have included three types of framing effects; however, this work only considered goal framing. Including attribute framing, which has also been examined in marketing, would be beneficial in future research. Second, this article pays attention to anchor prices related to organic food. However, it may also be worthwhile to explore whether an unrelated anchor value has the same effect on consumers’ responses.

## Data Availability Statement

The raw data supporting the conclusions of this article will be made available by the authors, without undue reservation.

## Ethics Statement

The studies involving human participants were reviewed and approved by the Ethics Committee of Jiangnan University. The patients/participants provided their written informed consent to participate in this study.

## Author Contributions

LW: conceptualization and writing – review and editing. HD: data curation and formal analysis. LS: validation. LS and HD: writing – original draft. All authors contributed to the article and approved the submitted version.

## Conflict of Interest

The authors declare that the research was conducted in the absence of any commercial or financial relationships that could be construed as a potential conflict of interest.
